# Phase 1 pilot study of e-mail support for people with long term conditions using the Internet

**DOI:** 10.1186/1472-6947-11-20

**Published:** 2011-04-05

**Authors:** Bryony Sheaves, Ray B Jones, Graham R Williamson, Rohan Chauhan

**Affiliations:** 1 Faculty of Health, University of Plymouth, Plymouth, PL4 8AA, UK; 2 Research & Development Department, Royal Devon & Exeter NHS Foundation Trust, Noy Scott House, Barrack Road, Exeter, EX2 5DW, UK

## Abstract

**Background:**

Use of the Internet for people with Long Term Conditions (LTCs) can have a positive effect on knowledge, social support, behavioural and clinical outcomes, yet there is concern that a 'digital divide' prevents some patients from benefitting. While some patients do not have access to the Internet, others that do may still lack expertise or the confidence to make full use of it. The aim of this pilot study was to develop an intervention and test methods for a definitive randomised controlled trial (RCT) of anonymous personal online email support for patients in this latter group.

**Methods:**

Recruitment success was evaluated by the number and appropriateness of participants recruited. A personalised e-health support intervention was developed. The provisional primary outcome was the extent to which the Internet affected the participants' confidence in dealing with their LTC. Primary outcome, seven process measures and two secondary outcomes measures were evaluated for completeness of data and sensitivity to detect changes.

**Results:**

Thirty nine participants were recruited, 29 after personally receiving a leaflet, seven via email advertising, and three via leaflets left in waiting areas. Most participants (61%) were aged over 60. The majority (21/38) rated themselves as experienced Internet users although only 5/38 had used discussion forums for their LTC. Piloting the intervention identified support needed as: (i) technical help with some websites, (ii) advice about issues such as anonymity, (iii) help in judging information quality, (iv) identification of relevant information (via 'Information Prescriptions'), (v) motivational support to try new sites. Attrition was fairly high: 20/39 completed follow up questionnaires. Three process measures showed ceiling effects and two had too many missing values to be useable.

**Conclusion:**

E-health support is a promising way of addressing the problems faced by older generation e-health seekers. Face-to-face leaflet distribution recruited sufficient numbers but additional locations other than hospital should be tried to recruit Internet novices with LTCs. An RCT is feasible and necessary to evaluate the potential benefits of anonymous email support. Our methods could be used by other researchers studying Internet use by people with LTCs.

## Background

Six out of 10 adults in England report a chronic health condition ranging from mild asthma to terminal cancer [1]. Given that British patients with such conditions account for 80% of all General Pracitioner visits and are twice as likely to be admitted to hospital than those without, this represents a major burden for health services as well as to the quality of life of patients themselves [1]. A recent review of the benefits and outcomes of digital health services found that people with LTCs can use the Internet for information, therapy, peer support and communication with clinicians, to achieve better health outcomes and better manage their care [2]. Examples include a web based information system that improved blood glucose control in type 2 diabetes patients [3], a computer mediated support group that improved emotional wellbeing and reduce negative mood in a group of women with breast cancer [4], and a personalised computer-based educational programme that reduced hospital admissions and improved morbidity in adults with asthma [5]. Recent systematic reviews [6,7] concluded that technological self-management systems could provide people with LTCs with a practical method of understanding and monitoring their condition, as well as therapeutic guidance to alter maladaptive behaviour.

Seventy percent of UK households were connected to the Internet in 2009 [8], with the over 65 age group starting to 'catch up' on younger age groups in the numbers using the Internet, but still only a minority (35%) compared to nearly 100% of 16-24 year olds having accessed the Internet. Many older users may lack the confidence to learn Internet skills through trial and error [9], unlike their younger counterparts who have grown up surrounded by such technology. Although recent empirical work has shown that even amongst younger people Internet expertise is far from universal [10]. These findings fit with other literature that suggests that difficulties with websites [11], lack of awareness of online health information and support, or being overwhelmed by the volume of different online methods [12] are all barriers to effective use. One way to overcome these barriers would be to offer the personal help of an experienced Internet user to help the less experienced population (those with lower 'Internet self-efficacy' [13]) with their health information-seeking needs.

Mead et al. [14] set up face-to-face e-health support at a general practice but few patients used the facility and the majority of those that did already had experience of using the Internet. A subsequent survey suggested that patients' confidence in their ability to use the technology was a potential barrier. Mead et al. [14] suggested that training interventions to enhance Internet self-efficacy might encourage online health behaviour, but acknowledged that concerns about privacy may have contributed to poor uptake of their designed intervention.

We think there is potential to provide e-health support to patients with LTCs via anonymous online email. Given that most interventions reporting benefit [2] are single condition and specific Internet applications, why provide support across all LTCs? Anecdotal evidence suggests that one barrier to wider implementation in the UK of e-health interventions, shown to be effective in a research studies, is concern about the 'digital divide'. It is not feasible to consider e-health support for single conditions for geographically limited area given the low numbers involved. On the other hand, if e-health support is offered to a wider geographical population recruitment has to be on a national basis so that face-face methods such as leaflet distribution are more difficult to assess. Finally, many people have co-morbidities and we prefer to take a more holistic rather than disease focused approach

We aim to carry out a definitive RCT of e-health support. The assumed model for the RCT is that by helping patients find and use Internet resources, they will (i) be able to make use of a wider range of Internet facilities and be more confident in that use, (ii) be more satisfied with the information and support that they get online for their LTC such that (iii) their use of the Internet will make them feel more in control of their LTC and make them more prepared to try new e-health services. This impact of the Internet on their feeling of control may also affect (iv) their overall feelings of control (self-efficacy) of the LTC and (v) their health status. If their clinician similarly makes use of the Internet and enables the patients to use the Internet and adjusts follow-up intervals [15] to take this into account, (vi) patients may have fewer health service visits and journeys. Hospitals and patients therefore could potentially save money. Patients may have improved self-efficacy and possibly health status, and be more able to participate in new clinician led e-health services. The e-health support intervention therefore aims to improve (i) and (ii) (process measures) leading to an improvement in (iii) (primary outcome), particularly for those who initially lacked Internet skills. Improvement in the primary outcome may lead to changes in (iv), (v), and (vi) but these are also influenced by many other factors.

However, little is known about how to identify patients that may benefit or exactly what form e-health support should take. Following the Medical Research Council's framework for complex interventions [16] we report here a phase 1 pilot study. This aimed to (i) test the efficiency of recruiting in hospital outpatients assessing the number and characteristics of patients who might benefit from the intervention (ii) determine what types of e-health support were needed, develop and document that support and its associated workloads, so that a consistent intervention can be trialled, and (iii) explore whether the outcome measures used are appropriate for a subsequent RCT.

## Methods

### Ethics

The study was reviewed and approved by the South West Research Ethics Committee of the National Health Service (NHS).

### Setting and raising awareness of the study

Patients were made aware of the project mainly through face-to-face contacts throughout the Royal Devon and Exeter Hospital NHS Foundation Trust in the summer of 2010. The hospital provides acute services to 350,000 people in Devon, England. It also offers specialist services such as cancer care, plastic and reconstructive surgery, orthopaedic surgery, paediatric care and renal services to people living further afield within the south west of England [17]. People in waiting and other areas were approached by BS, the study briefly explained, and leaflets with the study website address given to interested patients. If patients were not connected to the Internet, but their carer was, BS explained that carers could not participate *on behalf *of patients but could work *alongside *them helping with Internet access. Leaflets included unique study entry codes that indicated location within the hospital. Participants entered these codes into the study website at registration if they wished to take part. Additional methods for raising awareness included leaflets and posters left at some other hospital sites, emails to 13 outside patient support organisations, and emails to two local newspapers.

### Target audience, eligibility and recruitment

Our target audience was people with LTCs who had access to the Internet and an email account, but were novice or nervous users of the Internet and who thought that some help might be useful. We wanted to test the feasibility of recruiting 'anonymously' in hospital outpatient areas and wanted to know which patients this method would recruit, recognising that, by their very nature, patients who are nervous users of the Internet may be wary of joining a study to use it. Patients were eligible to join the study if: (i) they had a LTC (patients were given a list of examples but named their own LTC(s)), (ii) they had attended the hospital in the last two years for their LTC, and (iii) were aged 16 or older. A study website allowed potential participants to learn about the study by downloading information sheets and viewing an explanatory video. Those wishing to join the study and who met the eligibility criteria gave anonymous consent by entering their email address. Participants were encouraged to use anonymous email accounts.

### Baseline data

Following informed consent, BS emailed participants a hyperlink to an online questionnaire. This included all process and outcome measures amalgamated into one survey that participants completed at baseline and again following the e-health intervention. Additional file 1 shows the questions asked at baseline and Additional file 2 the follow up questions. Both surveys asked questions one at a time in a branching online questionnaire and were implemented using Limesurvey (http://www.limesurvey.org).

### Development of intervention and estimation of workloads

**Figure 1:**
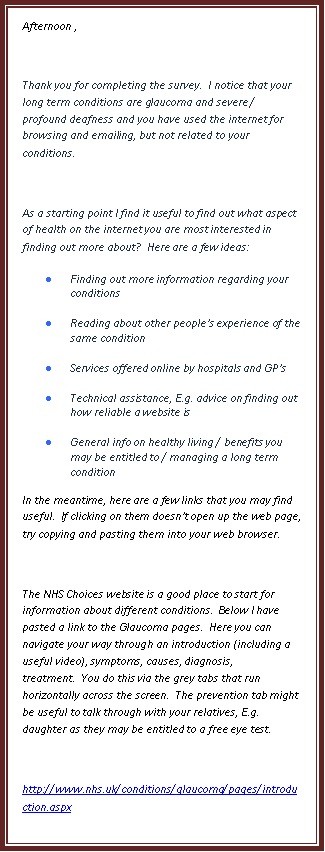
Example of first email

Following completion of the baseline survey, BS made email contact with participants. The initial email was personalised to each participant using data from the baseline survey such as satisfaction with information about their condition, confidence using the Internet, and uses of the Internet so far. BS aimed to encourage 'conversation', asking about Internet use for health needs, interest in reading about others' experiences of the same condition, need for technical assistance, and suggested links to relevant web pages (Figure 1).

Encouragement to comment was included in all emails. Mid-way through the e-health support intervention all participants were sent an email specifically requesting feedback on their experience of e-health support. Feedback emails were analysed thematically [18].

The time spent on e-health facilitation emails was recorded to estimate workloads.

### Process and Outcome Measures

The primary outcome measure being explored was the extent to which the Internet affected the participants' confidence in dealing with their long term condition. This was assessed via one question on a five point scale. Seven process measures were assessed: (i) the number of Internet applications used from browsing, email, instant messaging, Internet telephony, discussion forums, social networks, and other, (ii) the number of Internet applications used for their LTC, (iii) satisfaction with information obtained, (iv) understanding of information obtained, (v) utility of factual information about the LTC in the last month using the Internet (based on [19]), (vi) utility of Internet communication in the last month (asked in the same way as (v)), (vii) two questions that assessed Internet self-efficacy [13] on a scale from 2-20. Two secondary outcome measures were Lorig's self-efficacy scale which has been shown to have an internal consistency of 0.91 [20,21] and the SF-36 Health Status Questionnaire [22]. The SF-36 questionnaire has been shown to have criterion validity [23] and discriminant validity of the mental and physical functioning scales [24].

### Analysis

Ceiling effects occur if participants have scores at or near the top of the scale of measures at baseline and so are unlikely to improve. Floor effects are the converse when participants are unlikely to deteriorate. Various cut-offs have been used by others including maximum score [25], top 15% [26], top 20% [27], and top 25% [28]). We assessed the seven process and primary outcome measure for possible ceiling and floor effects by examining those in the top and bottom 25%.

We examined face validity of the outcome measures by assessing agreement between participant responses to the intervention emails (which were coded for signs of improvement on the outcome measures) and changes in the five outcome measures. For example, if a participant mentioned in an email response that they had tried a discussion forum, and had not previously done so, they were coded as having increased in their Internet uses. We would expect those who indicated such change to be more likely to show change on the quantitative measures.

The appropriateness of recruitment was assessed in two ways: (i) descriptive statistics were used to examine recruitment rates in relation to methods of recruitment, (ii) analysis of baseline outcome measures was used to assess whether we recruited people who could benefit from e-health support by examining 'room for improvement' on process and primary outcome measures.

## Results

### Recruitment

**Figure 2:**
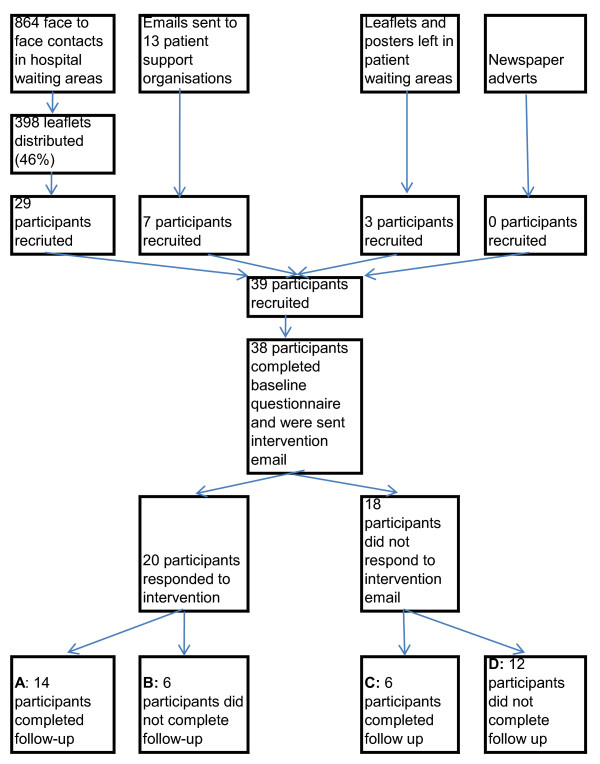
Patient flow diagram

Thirty nine participants were recruited over five weeks. A total of 864 people were approached about the study within the hospital, of these 398 (46%) took a leaflet and 29 signed up to the study via the study website (Figure 2). Recruitment following receipt of a leaflet varied from 0 to 12% between different hospital locations. Seven participants were recruited via email advertising to local patient groups (3 from Multiple Sclerosis Society and 4 from diabetes network). Three participants were recruited via leaflets and posters left in patient waiting areas without face-to-face contact with BS. Emails to local newspapers resulted in a short mention of the study in one newspaper but no resultant recruits.

### Characteristics of participants

Between the 39 participants that were recruited, a range of 35 different LTCs had been diagnosed, the most common of which were Diabetes (8), Multiple Sclerosis (4), Arthritis (3), Renal Failure (3), Prostate Cancer (3) and Heart Disease (3). Sixteen of the 39 participants had between 2 and 5 LTCs. Only two of the participants were newly diagnosed, within the last year. All participants were over 30 years of age and 23/38 (61%) aged over 60. (One person dropped out before completing the baseline questionnaire). About half were male (20/38), 28 lived more than 10 miles away and of these 12 lived more than 20 miles away. Participants had worse physical and mental health than the general UK population with means on the SF36 questionnaire of 33 and 47 respectively, compared to norms of 50. Sixteen had contacted their GP 2 or 3 times, and 5 four or more times in the last three months. Lorig's self-efficacy scale was completed by all participants and scores ranged from 1.7 to 9.8, mean 6.7, compared to the reference mean of 5.17 for 605 subjects with chronic disease [20].

### Prior use of the Internet and confidence in its use

Our participants rated themselves as fairly confident in Internet use using Barnoy's self-efficacy scale [13]; 21/38 rating themselves as 17/20 or greater. Yet, few had used discussion forums or social networks (Table 1).

**Table 1:** Uses of the Internet at baseline by 38 participants who completed baseline questionnaire.

Internet activity	For any purpose	For their Long Term Condition
**Browsing**	36	34
**Email**	36	9
**Instant Messaging**	7	0
**Internet telephony**	5	1
**Discussion Forums**	6	5
**Social Networks**	10	2

### Attrition

Twenty (51%) out of 39 participants completed both the baseline and follow up questionnaires (boxes A and C on Figure 2). There was no obvious relationship between attrition and source of recruitment, demographics, or Internet or condition self-efficacy. Most non-response occurred after completion of the baseline questionnaire and in (non) response to the first email. Four of 19 participants with incomplete data positively withdrew (rather than did not respond). One withdrew because his health was deteriorating and the remaining three for reasons related to interest in and utility of e-health support, for example *"I have not encountered any problems using the Internet and I already know all that I want to know about my MS".*

### Ceiling effects

Are the process and outcome variables appropriate for a subsequent RCT and did we recruit the 'right' participants? We can assess this to some degree by examining the distribution of scores on variables at baseline and the 'room for improvement'. Two of the process measures ((v) and (vi)) asked about Internet use in the last month; only 10 had obtained factual information and 8 used the Internet for communication about their LTC in the last month so we did not calculate utility scores [19]. Satisfaction, understanding, and Internet self-efficacy all had marked ceiling effects. Those not in the top 25% were thought to have 'room for improvement' (Table 2).

**Table 2:** Baseline distributions of five of the seven process measures and the primary outcome for 38 participants who completed the baseline survey (top row) and 19 who completed follow-up (second row).

Range of possible scores	Top 25% of scale	Middle 50% of scale	Bottom 25% of scale	All 38, or 19 completing follow-up
**Five of the seven process measures**				
**Internet applications (any purpose)**				
0-7	6	32	0	38
0-7	2	17	0	19
**Internet applications (LTC)**				
0-7	0	6	32	38
0-7	0	4	15	19
**Satisfaction with information obtained so far on LTC**				
1-5	14	21	3	38
1-5	7	11	1	19
**Understanding of information obtained so far on LTC**				
1-5	22	15	1	38
1-5	10	9	0	19
**Internet self-efficacy**				
2-20	21	16	1	38
2-30	13	6	0	19
**Primary outcome measure**				
**Internet makes me a lot more confident in dealing with LTC**				
1-5	6	32	0	38
1-5	4	15	0	19

### Appropriateness of recruitment

All participants exhibited room for improvement on the number of Internet applications used in relation to their LTC and at least one other variable. However, one participant only had room for improvement on Internet applications related to LTC and seven on that and one other measure. There was no obvious relationship between the source of recruitment and having room for improvement on more variables.

### Validity of quantitative measures in comparison with participant reports

Participant responses to intervention emails were coded for signs of improvement in the process and outcome measures. This classification of participant reports was compared with changes in four variables from baseline to follow up. Intention to treat analysis was used, i.e. all 39 participants recruited were included and those with no follow up data, coded as 'no change' from baseline. Based on very small numbers commenting, positive comments seemed to be related to improvements to scores on Internet use, satisfaction with information, and Internet self-efficacy, but not on the outcome scale of the Internet affecting confidence to manage their LTC (Table 3). However, as shown above, most scales suffered from ceiling effects; four participants who made positive comments for the outcome variable all scored 4 or 5/5 at baseline deteriorating by 1 or 2 points at follow up.

**Table 3:** Comparison of changes in scores by whether or not participants made positive comments in email exchanges.

Email comments	Number of participants	Mean score change from baseline to follow up
**Internet uses**		
Positive	5	1.20
None	34	0.41
**Internet self-efficacy**		
Positive	2	2.00
None	37	0.03
**Satisfaction with information**		
Positive	12	1.00
None	27	0.19
**Internet affecting confidence to manage LTC**		
Positive	4	-1.00
None	35	-0.06

### Development of the Intervention

As a result of continued learning throughout the study, a guide to e-health facilitation was developed. All support given to participants was personalised to their needs based on the information gained from the baseline survey as well as information disclosed in email exchanges. All support given was pitched at the participants' levels of Internet and health literacy. Internet literacy was partly assessed by stated Internet uses from the baseline questionnaire, but both Internet and health literacy were further explored by the personal email exchange with each participant. Five types of support that participants required were:

• Help to overcome technical difficulties in accessing the Internet or a particular website;

• Advice about different types of communication (issues of anonymity, social distance etc);

• Coaching to help them judge the quality of websites;

• Help in tailoring information via an 'Information Prescription';

• Motivational support in continuing to use sites or to try new types of site (e.g. discussion forum).

### General views about e-health support

Six participants responded to the email request for feedback with general positive regard to e-health support, for example, one participant commented: "E-health support is useful as it helps you to feel less isolated". Two participants commented on the nature of e-health support as being an impersonal method of support, for example, one participant commented: "I do so hope that it is an add-on rather than a replacement for personal service". Given that 47% of participants did not respond to an intervention email, when asked for feedback some participants commented with reasons. Themes that emerged from seven feedback emails were that e-health facilitation would be useful to others, or to that participant at another time. For example, one participant commented: "I think that I would have appreciated web site addresses when I was first diagnosed". Four participants provided feedback with regard to their motivation for joining the study, all four commented that they wanted new information and three of these same four commented that they wanted to help with research, for example, "I hoped to help by joining the study, and perhaps also to derive new information about diabetes".

Seventeen out of 20 participants reported that they thought email support would be useful to people such as them. In addition, 17/20 thought that there was a need for further research into e-health facilitation. A slightly lower number (12/20) considered that they might be able to see their clinician less frequently because of e-health services and qualitative comments suggested that this might be related to their diagnosis. For instance, a participant with glaucoma stressed the importance of regular eye checks that could not be replaced by online services. Fifteen of the twenty participants considered that their clinicians should be contactable via email, though concerns were raised about the immediacy of replies and the time needed for clinicians to be able to respond to queries. A majority of 16 out of 20 participants considered that the NHS should provide e-health support.

### Workload

Participants received an average of 2 intervention emails each with the mean total time spent on each patient of 76 minutes (range 5-260 minutes). This time included both time needed to research resources appropriate for that participant as well as to write the email itself. The average time is likely to decrease as experience and 'cut and paste' possibilities increase. Using these timings, we estimated that a sample of 600 participants was feasible for one research assistant for a phase 2 study.

## Discussion

The aims of the current study were to pilot e-health support for patients with LTCs who had access to the Internet but needed help in using it. In particular we aimed to (i) test recruitment, (ii) develop the e-health support intervention and (iii) explore the appropriateness of the outcome and process measures.

Studies that recruit known and named patients meeting particular clinical inclusion criteria to RCTs of alternative therapy expect recruitment rates of 70% or above. In a study of this nature, where recruitment is anonymous and the eligibility criteria (LTC, over 16, access to the Internet but who might benefit from help) have to be self-assessed we cannot know what proportion of those who were given leaflets met the eligibility criteria. We think that leaflet distribution to subsequently recruit 7% online is an efficient method but there may be concerns about 'self-selection'. On the other hand, there is a 'trade-off' between focused recruitment to achieve a higher recruitment rate, and inclusivity to ensure that all patients who may benefit from e-health support can participate.

On the other hand, more attention is needed to recruit participants who are novice Internet users and who can therefore benefit more from the intervention. Our exploration of 'room for improvement' indicated that most of the participants had high scores on self-reported Internet self-efficacy. Some of our participants joined the study 'to help'. Others indicated that e-health support would be more beneficial to them at a different time. More work is needed to ensure recruitment of those most in need of e-health support. Piloting in additional sites is needed: (i) General Practice where patients may have greater information-seeking needs being earlier in the trajectory of a condition, and (ii) novice computer classes for older people where participants are likely to have an LTC but also have low Internet self-efficacy. In both cases information given about the study needs to discourage altruistic patients who may not benefit personally from joining [29].

The 'room for improvement' analysis revealed that the majority of participants exhibited no room for improvement on the self-reported Internet self-efficacy measure. The participant population therefore exhibit a high degree of confidence in their Internet ability. However, these participants exhibited room for improvement on the number of Internet applications used in relation to their long term condition as well as many exhibiting room for improvement on Internet applications used in general. This suggests a lack of 'calibration' between Internet ability and feelings of confidence in using the Internet. This bias towards overconfidence in relation to actual ability to complete a task has been recognised and experimentally manipulated before [30-32]. We will revise the order and exact way in which questions are asked to get a more integrated and consistent measure of Internet ability and self confidence (questionnaire available from authors).

Most participants thought that e-health support would be useful. Given that the support offered was personalised and directed mainly by the participants' needs, this study has begun to uncover some of the barriers facing older patients who seek to make use of online health resources. A guide on providing e-health support has been produced indicating five types of support: (i) technical help with some websites, (ii) advice about issues such as anonymity, (iii) help in judging information quality, (iv) identification of relevant information (via 'Information Prescriptions'), (v) motivational support to try new sites.

The guide includes examples of how email exchange might help tailor advice to individuals. We made it clear to participants that the e-health facilitator did not provide health information, but advised participants on how to use the Internet for health information and support, and encouraged them to do so. Further work is needed to ensure consistency in the assessment of Internet and health literacy of the participants between multiple e-health facilitators.

The basic Internet skill needed to join our study was to be able to use email. The majority of our users thought that clinicians should be contactable by email. A recent systematic review [33] of 24 mainly US studies concluded that while benefits of e-mails in enhancing communication were recognized by both patients and providers, concerns about confidentiality and security were also expressed. Use of the Internet to communicate with a known health professional is still rare in Europe [34]. Others have suggested that a more structured approach using computer-patient interviewing [35] is the way forward. The evaluation of peer-support discussion groups has methodological difficulties [36] and the evidence as to their benefit in health outcomes is limited [37,38] but users of such forums generally perceive there to be benefit [39]. However, few of our participants had previously used discussion forums. E-health support could help novice users to make use of them.

Attrition was high, with half of the participants who registered with the study not completing follow up. Although, this is comparable with other web based health interventions [40-44], an RCT needs to consider ways to engage and sustain both intervention and control participants. On reflection, some of the measures used, such as the SF-36, provided outcome assessment of only secondary importance and were relatively time consuming for participants. Although, we cannot know the reasons for non-response at follow up, others have suggested the burden of data collection may often be responsible [45]. Attrition may be reduced by collecting only complete, accurate, and valid primary outcome measures and dropping secondary outcomes such as health status for this study. The first email of the intervention needed to be more engaging. Reducing the proportion of data collection at baseline used for outcome assessment and increasing data collection to enable tailoring of subsequent intervention emails, may help reduce attrition.

Comparison between coding of email dialogue and quantitative measures from the questionnaires revealed significant correspondence for two variables, Internet uses, and satisfaction with information. This same correspondence between quantitative and qualitative data was not found for the extent to which the Internet affects the participants' confidence in managing their long term condition, nor their self-efficacy for managing their long term condition. However, the population mean for these latter two variables showed a decrease in performance from baseline to the follow up questionnaire and further investigation is needed to ascertain why there is a lack of correspondence for these measures when compared to participants' comments.

### Limitations

Our approach to e-health support will not help patients who have no access to the Internet. The conclusions drawn regarding the appropriateness of the recruitment methods, intervention and outcome measures are based on results derived from a sample of 39 participants who had high Internet self-efficacy scores. The validity of these conclusions beyond this small, Internet confident sample cannot be certain. Further piloting in primary care is needed.

## Conclusions

Chronic diseases account for under 3% of conditions but half of bed day use [1]. Given the rapid developments in web 2.0 technologies, the Internet has potential to ease this burden by aiding self-management skills. To make this effective and equitable we may need to provide e-health support. This pilot study suggests an RCT is feasible and should be undertaken after further piloting. Our findings with respect to the methods used are of use to other researchers.

## List of Abbreviations

LTC: Long Term Condition; RCT: Randomised Controlled Trial; NHS: National Health Service.

## Competing interests

The authors declare that they have no competing interests.

## Authors' contributions

RBJ: had the idea for the study, applied and secured funding, gained ethical approval, set up the web recruitment, designed the questionnaires, managed the project on a day to day basis, contributed to the literature review, edited and submitted the paper and is the corresponding author. BS: ran the recruitment and intervention on a day to day basis, contributed to the literature review, and initially took the lead on writing the paper. GRW: was co-grant holder, managed the project on a day to day basis, facilitated local contacts, edited the paper. RC: was co-grant holder, helped manage the project, facilitated local contacts and considerations, contributed ideas to dissemination and embedding of e-health support, edited the paper. All authors have read and agreed the final manuscript.

## Pre-publication history

The pre-publication history for this paper can be accessed here:

http://www.biomedcentral.com/1472-6947/11/20/prepub

## Supplementary Material

Additional file 1 baseline survey.mht Click here for file

Additional file 2 follow up survey.mht Click here for file
